# Supplementary material for: Smoothies Marketed in Spain: Are They Complying with Labeling Legislation?
Source: Nutrients. 2023 Oct 18;15(20):4426. doi: 10.3390/nu15204426 (PMC10610167; doi:10.3390/nu15204426)
Supplement: Supplementary file 1 [file nutrients-15-04426-s001.zip › nutrients-2644766-supplementary.pdf]

Table S1. Degree of compliance with legal requirements for mandatory food information in smoothies labels with fruits or with fruits and vegetables. V: complying legislation; X: not complying; -: not present in the label.

Smoothie Code	NF	Ingredients List							Qn	SL	CU	BA	CO	IU	ND	MA	Total	
		S	Ad	Ar	Ci	Qi	Ai	VM									V	X
Smoothies with fruits																		
**F1**	X	V	-	-	-	X	-	-	V	V	V	V	-	-	V	-	5	2
**F2**	V	V	-	-	-	V	-	V	V	V	V	V	-	V	X	V	10	1
**F3**	X	V	-	-	-	V	-	V	V	V	V	V	-	-	X	-	7	2
**F4**	X	V	-	-	-	V	-	V	V	V	V	V	-	V	X	V	9	2
**F5**	X	V	-	-	-	V	-	V	V	V	V	V	-	V	X	-	8	2
**F6**	V	V	-	-	-	V	-	-	V	V	V	V	-	V	V	V	10	0
**F7**	V	V	-	-	-	V	-	V	V	V	V	V	-	V	X	V	10	1
**F8**	V	V	V	V	-	V	-	V	V	V	V	V	-	V	V	-	12	0
**F9**	V	V	-	-	-	V	-	V	V	V	V	V	-	V	X	V	10	1
**F10**	V	V	-	-	-	V	-	V	V	V	V	V	-	V	X	V	10	1
**F11**	X	V	V	-	-	V	-	-	V	V	V	V	-	V	V	-	9	1
**F12**	X	V	V	-	-	V	-	-	V	V	V	V	-	V	V	-	9	1
**F13**	V	V	-	-	-	V	-	-	V	V	V	V	-	V	V	V	10	0
**F14**	V	V	-	-	-	V	-	V	V	V	V	V	-	V	X	V	10	1
**F15**	V	V	V	-	-	V	-	V	V	V	V	V	-	-	V	-	10	0
**F16**	V	V	-	-	-	V	-	-	V	V	V	V	-	-	V	-	8	0
**F17**	X	V	-	-	-	V	-	V	V	V	V	V	-	V	X	V	9	2
**F18**	V	V	-	-	-	V	-	V	V	V	V	V	-	-	X	-	8	1
**F19**	X	V	V	-	-	V	-	-	V	V	V	V	-	V	V	-	9	1
**F20**	X	V	-	-	-	V	-	-	V	V	V	V	-	-	V	V	8	1
**F21**	V	V	-	-	-	V	-	V	V	V	V	V	-	V	X	V	10	1
**F22**	V	V	-	-	-	V	-	V	V	V	V	V	-	V	X	V	10	1
**F23**	V	V	-	-	-	V	-	V	V	V	V	V	-	V	X	V	10	1
**F24**	V	V	V	-	-	V	-	-	V	V	V	V	-	-	V	-	9	0
**F25**	X	V	-	-	-	X	-	V	V	V	V	V	-	V	X	V	8	3
**F26**	X	V	-	-	-	V	-	V	V	V	V	V	-	V	X	V	9	2
**F27**	X	V	V	V	-	V	-	-	V	V	V	V	-	-	V	-	9	1
**F28**	V	V	-	V	-	V	-	V	V	V	V	V	-	-	X	-	9	1
**F29**	X	V	-	-	-	V	-	-	V	V	V	V	-	-	V	-	7	1
Smoothies with fruits and vegetables																		
**FV1**	X	V	-	-		V	-	-	V	V	V	V	-	V	V	V	9	1
**FV2**	X	V	-	-	-	V	-	-	V	V	V	V	-	V	X	V	8	2
**FV3**	V	V	-	-	-	V	-	V	V	V	V	V	-	-	X	-	8	1
**FV4**	V	V	V	V	-	V	-	V	V	V	V	V	-	V	X	V	12	1
**FV5**	X	V	-	-	-	V	-	-	V	V	V	V	-	-	V	-	7	1
**FV6**	X	V	-	-	-	V	-	-	V	V	V	V	-	-	V	-	7	1

NF: Name of the food. S: Simple ingredients. Ci: Compound ingredients. Ad: Additives. Ar: Aromas. Qi: Quantity of certain ingredients. Qn: Net quantity of the food. Ai: Allergens/intolerances. VM: Vitamins and/or minerals. SL: Shelf life. CU: Conservation/use. BA: Business name and address. CO: Origin country. IU: Instructions for use. ND: Nutrition claims. MA: Modified atmosphere packaging.

Table S2. Degree of compliance with legal requirements for mandatory food information in smoothies labels with fruits and other ingredients. V: complying legislation; X: not complying; -: not present in the label.

Smoothie Code	NF	Ingredients List							Qn	SL	CU	BA	CO	IU	ND	MA	Total	
		S	Ad	Ar	Ci	Qi	Ai	VM									V	X
Smoothies with fruits and dairy products																		
**FD1**	X	V	V	-	-	V	-	-	V	V	V	V	-	V	V	-	9	1
**FD2**	V	V	-	V	-	V	V	-	V	V	V	V	-	-	V	-	10	0
**FD3**	V	V	-	-	-	V	X	-	V	V	V	V	-	-	-	-	7	1
**FD4**	V	V	X	V	-	V	V	V	V	V	V	V	-	-	V	-	11	1
Smoothies with fruits, vegetables and dairy products																		
**FVD1**	X	V	V	-	-	V	-	-	V	V	V	V	-	-	V	-	8	1
**FVD2**	X	V	-	-	-	V	X	-	V	V	V	V	-	-	-	-	6	2
Smoothies with fruits and cereals																		
**FC1**	V	V	-	-	-	V	-	V	V	V	V	V	-	-	X	-	8	1
**FC2**	X	V	-	-	V	V	V	V	V	V	V	V	-	V	X	V	11	2
**FC3**	X	V	-	-	V	V	V	V	V	V	V	V	-	V	X	V	11	2
**FC4**	X	V	-	-	V	V	V	V	V	V	V	V	-	V	X	V	11	2
**FC5**	V	V	-	-	-	V	-	-	V	V	V	V	-	V	V	V	10	0
**FC6**	V	V	-	-	-	V	-	-	V	V	V	V	-	V	V	V	10	0
Smoothies with fruits, vegetables and cereals																		
**FVC1**	X	V	-	-	-	V	-	V	V	V	V	V	-	-	X	-	7	2
**FVC2**	V	V	-	-	-	V	-	V	V	V	V	V	-	-	X	-	8	1
Smoothies with fruits, cereals and dairy products																		
**FCD1**	V	V	V	V	-	V	V	-	V	V	V	V	-	-	V	-	11	0
**FCD2**	V	V	V	V	-	V	V	-	V	V	V	V	-	-	V	-	11	0
**FCD3**	V	V	V	V	-	V	V	V	V	V	V	V	-	-	V	V	13	0
**FCD4**	V	V	-	-	-	V	V	-	V	V	V	V	-	V	V	-	10	0
**FCD5**	X	V	-	V	V	V	V	-	V	V	V	V	-	V	V	V	12	1
Smoothies with fruits, vegetables, cereals and dairy products																		
**FVCD1**	X	V	-	V	-	V	V	-	V	V	V	V	-	V	V	V	11	1
**FVCD2**	X	V	-	V	-	V	V	-	X	V	-	V	-	V	V	-	8	2
**FVCD3**	V	V	V	-	-	V	V	V	V	V	V	V	-	V	X	-	11	1

NF: Name of the food. S: Simple ingredients. Ci: Compound ingredients. Ad: Additives. Ar: Aromas. Qi: Quantity of certain ingredients. Qn: Net quantity of the food. Ai: Allergens/intolerances. VM: Vitamins and/or minerals. SL: Shelf life. CU: Conservation/use. BA: Business name and address. CO: Origin country. IU: Instructions for use. ND: Nutrition claims. MA: Modified atmosphere packaging.

Table S3. Degree of compliance with legal requirements for optional food information in smoothies labels with fruits and smoothies with fruits and vegetables. V: complying legislation; X: not complying; -: not present in the label.

Smoothie Code	Nutritional claims																		L	Qo	Total	
	1	2	3	4	5	6	7	8	9	10	11	12	13	14	15	16	17	18			V	X
Smoothies with fruits																						
**F1**	V	V	-	-	V	-	V	-	-	-	-	-	-	-	-	-	-	-	V	-	5	0
**F2**	V	V	V	-	-	-	-	V	-	-	-	-	-	-	-	-	-	-	V	-	5	0
**F3**	V	-	-	-	-	V	V	V	-	-	V	V	-	-	-	-	-	-	V	-	7	0
**F4**	-	-	-	-	-	V	-	-	-	-	-	-	-	-	-	-	-	-	V	-	2	0
**F5**	V	-	-	-	-	V	-	V	-	-	V	-	V	-	-	-	-	-	V	V	7	0
**F6**	-	-	-	-	-	V	-	-	-	-	V	V	-	-	-	-	-	-	V	-	4	0
**F7**	V	-	-	-	-	V	-	-	-	-	V	-	V	-	-	-	-	-	V	V	6	0
**F8**	-	-	-	-	-	-	-	-	-	-	V	-	-	-	-	-	-	-	V	-	2	0
**F9**	V	V	V	-	-	V	-	X	-	-	-	-	-	-	-	-	-	-	V	-	5	1
**F10**	V	V	V	-	-	V	-	X	-	-	-	-	-	-	-	-	-	-	V	-	5	1
**F11**	-	-	-	-	-	-	-	-	-	-	-	-	-	-	-	-	-	-	V	-	1	0
**F12**	-	-	-	-	-	-	-	-	-	-	-	-	-	-	-	-	-	-	V	-	1	0
**F13**	V	V	V	-	-	V	V	-	-	-	V	-	-	-	-	-	-	-	V	-	7	0
**F14**	V	V	V	-	-	V	-	X	-	-	V	-	-	-	-	-	-	-	V	-	6	1
**F15**	V	-	-	-	-	V	V	-	-	-	V	V	-	-	-	-	-	-	V	-	6	0
**F16**	V	V	-	-	V	-	V	-	-	-	-	-	-	-	-	-	-	-	V	-	5	0
**F17**	V	-	-	-	-	-	-	V	X	-	V	-	-	-	-	-	-	-	V	-	4	1
**F18**	V	-	-	-	-	V	-	X	-	-	V	-	V	-	-	-	-	-	V	V	6	1
**F19**	-	-	-	-	-	-	-	-	-	-	-	-	-	-	-	-	-	-	V	-	1	0
**F20**	V	V	-	-	-	V	V	-	-	-	V	-	-	-	-	-	-	-	V	-	6	0
**F21**	V	V	V	-	-	V	-	V	-	-	-	-	-	-	-	-	-	-	V	-	6	0
**F22**	V	V	V	-	-	V	-	V	-	-	-	V	-	-	-	-	-	-	V	-	7	0
**F23**	-	-	-	-	-	V	-	-	-	-	V	-	-	-	-	-	-	-	V	-	3	0
**F24**	-	-	-	-	-	-	-	-	-	-	V	-	-	-	-	-	-	-	V	-	2	0
**F25**	V	-	-	-	-	V	-	-	-	-	V	-	-	-	-	-	-	-	V	-	4	0
**F26**	V	-	-	-	-	V	-	-	-	-	V	V	-	-	-	-	-	-	V	-	5	0
**F27**	V	-	-	-	-	V	V	-	-	-	V	V	-	-	-	-	-	-	V	-	6	0
**F28**	V	V	V	-	V	-	-	V	-	-	V	-	-	-	-	V	-	-	V	-	8	0
**F29**	V	V	-	-	-	-	V	-	-	-	V	-	-	-	-	-	-	-	V	-	5	0
Smoothies with fruits and vegetables																						
**FV1**	V	V	V	-	-	V	V	-	-	-	V	-	-	-	-	-	-	-	V	-	7	0
**FV2**	V	-	-	-	-	V	-	-	-	-	V	V	-	V	-	-	-	-	V	-	5	0
**FV3**	-	-	-	-	-	-	V	X	-	-	V	-	-	-	X	-	-	-	V	-	3	2
**FV4**	-	-	-	-	-	V	-	-	-	-	V	-	-	V	-	-	-	-	V	-	4	0
**FV5**	V	V	-	-	-	-	V	-	-	-	V	-	-	-	-	-	-	-	V	-	5	0
**FV6**	V	V	-	-	-	-	V	-	-	-	V	-	-	-	-	-	-	-	V	-	5	0

1: without added sugars or 0% added sugars. 2: without preservatives. 3: without colorants. 4: without artificial aromas. 5: 100% natural. 6: % fruit. 7: bio, eco. 8: source of vitamin C, with vitamin C. 9: source of fiber. 10: rich in calcium, source of calcium, with calcium. 11: without gluten. 12: without lactose. 13: without milk, powder milk or added cream. 14: without palm oil. 15: antioxidant, detox, relaxing or energetic.

Table S4. Degree of compliance with legal requirements for optional food information in smoothies labels with other ingredients. V: complying legislation; X: not complying; -: not present in the label.

Smoothie Code	Nutritional claims																		L	Qo	Total	
	1	2	3	4	5	6	7	8	9	10	11	12	13	14	15	16	17	18			V	X
Smoothies with fruits and dairy products																						
**FD1**	-	-	-	-	-	-	-	-	-	-	-	-	-	-	-	-	-	-	V	-	1	0
**FD2**	-	V	V	V	V	-	-	-	-	V	V	-	-	-	-	-	V	V	V	V	10	0
**FD3**	V	V	V	-	-	-	V	-	-	-	V	-	-	-	-	-	-	-	V	-	6	0
**FD4**	-	-	-	-	-	-	-	-	-	V	-	-	-	-	-	-	-	-	V	V	3	0
Smoothies with fruits, vegetables and dairy products																						
**FVD1**	-	-	-	-	-	-	-	-	-	-	-	-	-	-	-	-	-	-	V	-	1	0
**FVD2**	V	V	V	-	-	-	V	X	-	-	V	-	V	-	-	-	-	-	V	-	7	1
Smoothies with fruits and cereals																						
**FC1**	-	-	-	-	-	-	V	V	-	-	V	V	-	-	X	-	-	-	V	-	5	1
**FC2**	V	V	V	-	-	-	-	V	-	-	V	-	-	-	-	-	-	-	V	-	6	0
**FC3**	V	-	-	-	-	-	-	-	X	-	-	-	-	-	-	-	-	-	V	-	3	0
**FC4**	V	-	-	-	-	V	-	-	-	-	-	-	-	V	-	-	-	-	V	-	4	0
**FC5**	V	-	-	-	-	-	-	-	-	-	-	-	-	-	-	-	-	-	V	-	2	0
**FC6**	V	-	-	-	-	-	-	X	-	-	-	-	-	-	-	-	-	-	V	-	2	1
Smoothies with fruits, vegetables and cereals																						
**FVC1**	-	-	-	-	-	-	V	X	-	-	V	V	-	-	X	-	-	-	V	-	4	2
**FVC2**	-	-	-	-	-	-	V	-	-	-	V	V	-	-	X	-	-	-	V	-	4	1
Smoothies with fruits, cereals and dairy products																						
**FCD1**	-	-	-	-	-	-	V	-	-	-	V	-	-	-	-	-	-	-	V	-	3	0
**FCD2**	-	-	-	-	-	-	V	-	-	-	V	-	-	-	-	-	-	-	V	-	3	0
**FCD3**	-	V	V	X	X	-	-	-	-	V	-	-	-	-	-	-	-	-	V	-	4	2
**FCD4**	V	-	-	-	-	-	V	-	-	-	-	-	-	-	-	-	-	-	V	-	3	0
**FCD5**	-	-	-	-	-	V	-	-	-	-	-	-	-	V	-	-	-	-	V	-	3	0
Smoothies with fruits, vegetables, cereals and dairy products																						
**FVCD1**	V	-	-	-	-	V	-	-	-	-	V	-	-	V	-	-	-	-	V	-	5	0
**FVCD2**	V	-	-	-	-	V	-	-	-	-	V	-	-	V	-	-	-	-	V	-	5	0
**FVCD3**	V	-	-	-	-	V	V	-	-	-	-	-	-	-	-	-	-	-	V	-	4	0

1: without added sugars or 0% added sugars. 2: without preservatives. 3: without colorants. 4: without artificial aromas. 5: 100% natural. 6: % fruit. 7: bio, eco. 8: source of vitamin C, with vitamin C. 9: source of fiber. 10: rich in calcium, source of calcium, with calcium. 11: without gluten. 12: without lactose. 13: without milk, powder milk or added cream. 14: without palm oil. 15: antioxidant, detox, relaxing or energetic.

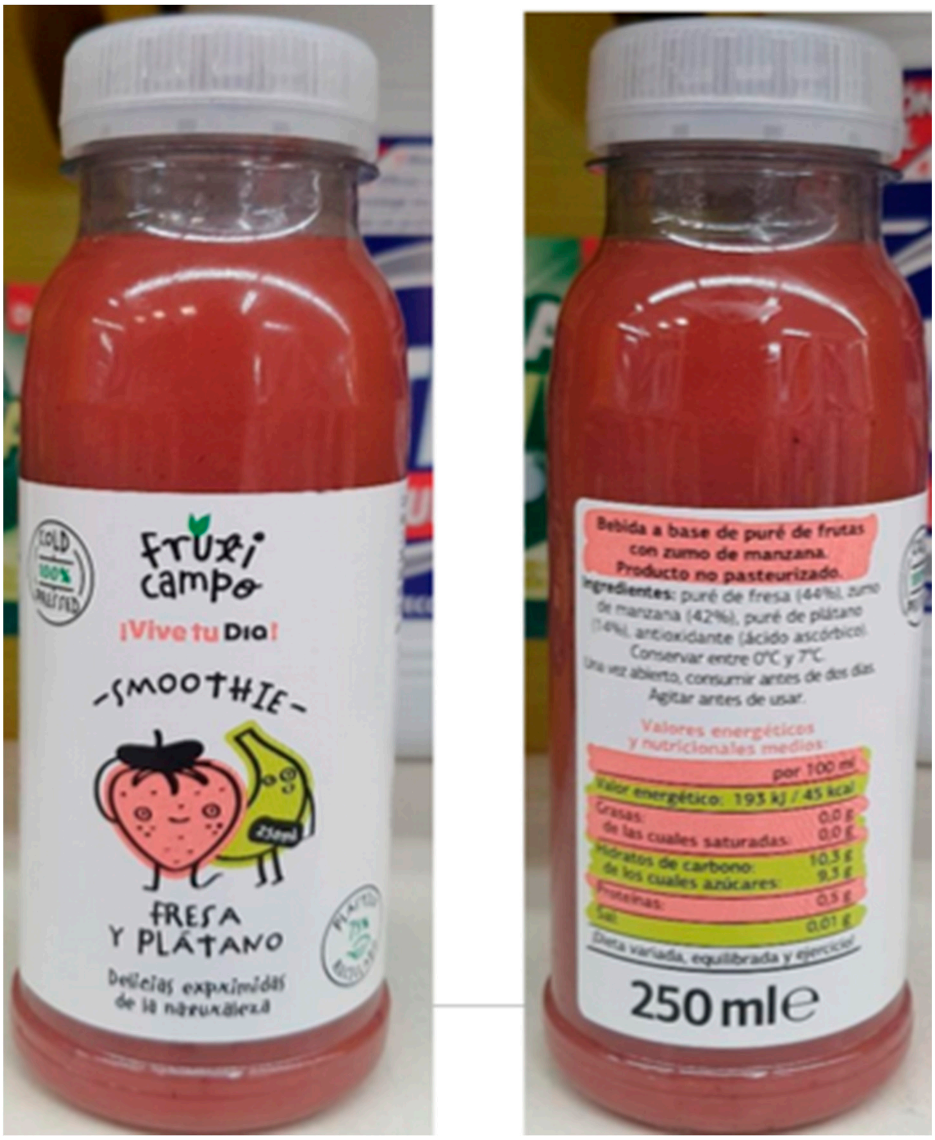

Figure S1. Smoothie example, type F (with fruits).
